# Effect of Different Ratios of Blue and Red LED Light on Brassicaceae Microgreens under a Controlled Environment

**DOI:** 10.3390/plants10040801

**Published:** 2021-04-19

**Authors:** Aušra Brazaitytė, Jurga Miliauskienė, Viktorija Vaštakaitė-Kairienė, Rūta Sutulienė, Kristina Laužikė, Pavelas Duchovskis, Stanisław Małek

**Affiliations:** 1 Lithuanian Research Centre for Agriculture and Forestry, Institute of Horticulture, Kaunas str. 30, LT-54333 Babtai, Lithuania; jurga.miliauskiene@lammc.lt (J.M.); Viktorija.Vastakaite-Kairiene@lammc.lt (V.V.-K.); Ruta.Sutuliene@lammc.lt (R.S.); Kristina.Lauzike@lammc.lt (K.L.); Pavelas.duchovskis@lammc.lt (P.D.); 2 Department of Ecology and Silviculture, Faculty of Forestry, University of Agriculture in Krakow, 31-425 Krakow, Poland; stanislaw.malek@urk.edu.pl

**Keywords:** microgreens, blue light, red light, LED, mineral elements

## Abstract

The consumption of microgreens has increased due to their having higher levels of bioactive compounds and mineral nutrients than mature plants. The lighting conditions during the cultivation of microgreens, if optimally selected, can have a positive effect by further increasing their nutritional value. Thus, our study aimed to determine the changes in mineral nutrients contents of Brassicaceae microgreens depending on different blue–red (B:R) light ratios in light-emitting diode (LED) lighting and to evaluate their growth and nutritional value according to different indexes. Experiments were performed in controlled environment growth chambers at IH LRCAF, 2020. Microgreens of mustard (*Brassica juncea* ‘Red Lace’) and kale (*Brassica napus* ‘Red Russian’) were grown hydroponically under different B:R light ratios: 0%B:100%R, 10%B:90%R, 25%B:75%R, 50%B:50%R, 75%B:25%R, and 100%B:0%R. A 220 μmol m^−2^ s^−1^ total photon flux density (TPFD), 18 h photoperiod, 21/17 ± 2 °C temperature and 60% ± 5% relative humidity in the growth chamber were maintained during cultivation. We observed that an increasing percentage of blue light in the LED illumination spectrum during growth was associated with reduced elongation in the microgreens of both species and had a positive effect on the accumulation of mostly macro- and micronutrients. However, different B:R light ratios indicate a species-dependent response to changes in growth parameters such as leaf area, fresh and dry mass, and optical leaf indexes such as for chlorophyll, flavonol, anthocyanin, and carotenoid reflectance.

## 1. Introduction

In the past few decades, people have become more interested in their health and green eating and have increased their consumption of vegetables, which are sources of various health-beneficial compounds, including mineral elements. Humans require certain mineral nutrients in large amounts, while others are necessary in trace quantities, and higher concentrations can be detrimental [1,2]. Macronutrients are essential to humans as cofactors of vitamins and enzymes, are vital for electrical signaling in nerves, and are necessary for optimal teeth and bone health. Various micronutrients serve as cofactors for numerous enzymes that are necessary for different metabolic processes and antioxidant activity, in addition to being essential for the immune system [3,4,5]. More than half of the world’s population is malnourished because of the insufficient mineral nutrient content in their food [6]. One way to increase mineral concentration in plants is through biofortification. This was proposed as a solution to reduce mineral malnutrition not only through breeding and biotechnology but also through a variety of agronomic practices, including artificial lighting, which is used for vegetable cultivation in controlled environment agriculture (CEA) [2,7,8].

The application of the ecologically friendly technology of light-emitting diode (LED) lighting in CEA has many advantages, such as the ability to select light wavelengths, change intensity, and reduce energy costs [9]. LED technology has been used to regulate light quality and quantity and thus influence plant growth and development in addition to indicate the physiological response to photooxidative changes by altering nutrients in vegetables [9,10,11,12,13,14,15,16,17,18]. Many studies are related to plant cultivation under LEDs of blue (B) and red (R) light as they have the highest photon efficiency. It is well known that such lights are better absorbed by chlorophylls than the light of other wavelengths in the visible spectrum [9,18]. Red light has been reported to promote photosynthesis and growth by increasing plant height, fresh and dry weight, and leaf area [19,20]. Blue light affects chlorophyll concentrations, photomorphogenesis, stomatal opening, and antioxidant accumulation [21,22]. However, data in the literature show that an appropriate B:R light ratio may be more favorable for photosynthesis and the content of various bioactive compounds in plants [20,23,24]. However, there is still a lack of information on how manipulation of the light spectrum affects changes in the mineral element content of various leafy vegetables, and the available information is contradictory. Some studies have demonstrated the positive effect of short-term monochromatic blue light or its percentage in different light spectrum compositions on the mineral element content in plants [25,26,27,28,29]. Some authors have reported that red LED or its higher percentage in blue–red lighting increased some mineral nutrients in marigold [30], lettuce [31], and basil [24]. On the other hand, when assessing the effect of blue or red light, or their ratio, on mineral elements, the focus is on their content in the edible part, regardless of the effects of lighting on their uptake from the solution or substrates to the roots, or from the roots to the shoots of leafy vegetables, including such specialty crops as microgreens.

Microgreens are seedlings of vegetables, herbs, or even wild species that are grown to the stage of fully opened cotyledons and vary in flavor, color, and texture [15]. The consumption of microgreens has increased due to their higher levels of bioactive compounds and mineral nutrients compared with mature plants. Although about 100 plant species can be cultivated as microgreens, Brassicaceae plants such as arugula, broccoli, cabbage, kale, and mustard are the most popular choices due to their easy germination, short growing time, and variety in flavor and color [5,32]. The role of Brassicaceae microgreens in improving health can be attributed to their high levels of bioactive compounds such as ascorbic acid, carotenoids, tocopherols, and phenolic compounds in addition to glucosinolates and mineral nutrients [33]. Having a wide range of possible cultivation environments ranging from open fields to indoor environments makes them attractive for growth in individual households on a small scale as well as on a large scale for commercial purposes by industries [34]. Indoor cultivation allows year-round production of microgreens and the manipulation of light to improve plants’ nutritional qualities [14,15,17,18]. We hypothesized that an optimal blue–red light ratio would have a positive effect on the content of mineral nutrients without adversely affecting their yield and nutritional value. Thus, our study aimed to determine the changes in mineral elements of Brassicaceae microgreens depending on different blue–red light ratios in light-emitting diode (LED) lighting and to evaluate their growth and nutritional value via numerous indexes.

## 2. Results

### 2.1. Mustard

The results from assaying mineral content show that having a higher percentage of blue light (B50–B100) in the illumination spectrum during cultivation resulted in a higher content of mineral nutrients in mustard microgreens, with the exception of nitrogen (N) (Table 1 and Table 2). However, differences in the various mineral nutrients were found depending on the light ratio. The B50R50, B75R25, and B100R0 treatment resulted in a significantly higher content of phosphorus (P), magnesium (Mg), sulfur (S) (Table 1), and manganese (Mn) (Table 2) in comparison with treatments with a lower percentage of blue light (B0R100, B10R90 and B25R75). Calcium (Ca) content increased under B50R50 and B75R25 lighting. The B100R0 treatment resulted in the highest content of potassium (K) (Table 1). The accumulation of iron (Fe) and copper (Cu) was mostly enhanced by B75R25 and B100R0, and zinc (Zn) was enhanced only by B75R25. Different lighting had no effect on boron (B) content in mustard microgreens, except for the B25R75 treatment, where the content was significantly lower (Table 2).

The bioconcentration factor (BCF) was calculated to evaluate the capability of mustard microgreens to extract and accumulate mineral nutrients in roots (Table S1). B25R75 increased the BCF of most mineral nutrients, except Mn, Fe, and Cu, of which the BCF was higher under B50R50.

The capability of mustard microgreens to accumulate mineral nutrients in the aboveground tissue was established by calculating the translocation factor (TF). There was a tendency for higher TF for most mineral nutrients under a higher percentage (B50R50, B75R25, and/or B100R0) and lower TF under a lower percentage (B0R100, B10R90, and/or B25R75) of B LED lighting (Table S2).

**Figure 1:**
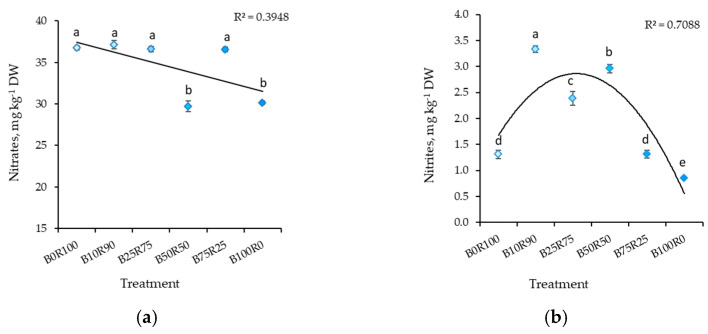
Effect of different ratios of blue and red LED lighting on the nitrates (**a**) and nitrites (**b**) of mustard microgreens. B0R100, B10R90, B25R75 B50R50, B75R25, and B100R0: indicate the percentage of blue (B) and red (R) light. All values in the table are expressed as mean ± standard error (*n* = 3). Means with different letters are significantly different at the *p* < 0.05 level according to Tukey’s honestly significant difference test.

The LED lighting’s spectral composition significantly influenced the nitrate and nitrite accumulation in mustard. B50R50 and B100R0 resulted in significantly lower nitrate content in mustard microgreens (Figure 1a). The lowest nitrite content was found in plants grown under B100R0. B75R25 and B0R100 treatments also resulted in significantly lower nitrite contents (2.5 times) compared to plants grown under B10R90, for which the highest nitrite content was measured of all light treatments (Figure 1b).

By increasing the B light percentage, the hypocotyl length of mustard microgreens decreased (Table 3). However, monochromatic red (B0R100) and blue (B100R0) light resulted in significant hypocotyl elongation. B100R0 caused an increase in root length, leaf area, shoot fresh and dry weight, and shoot-to-root ratio. Meanwhile, B50R50 and B75R25 lighting had a contrary effect on growth parameters, except for root length, which was shorter under B10R90. On the other hand, the shoot fresh and dry weight of mustard microgreens under lighting with a lower percentage of blue light (B0R100, B10R90, and B25R75) was similar to that under B100R0 treatment. Differing B:R did not affect root fresh and dry weight.

**Figure 2:**
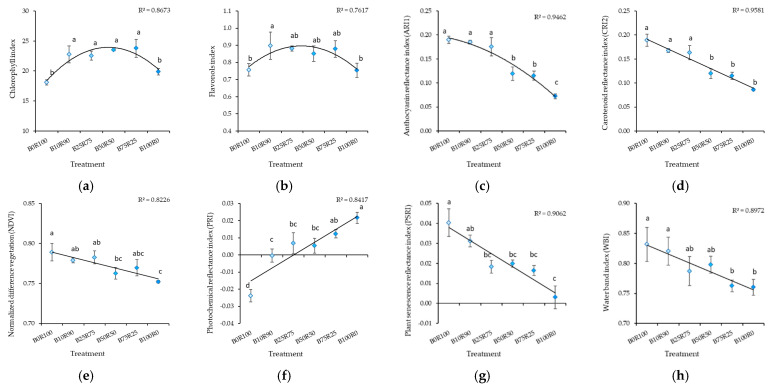
Effect of different blue–red light ratios in LED lighting on the chlorophyll (CHL) (**a**), flavonols (FLA) (**b**), anthocyanin reflectance (ARI1) (**c**), carotenoid reflectance (CRI2) (**d**), normalized difference vegetation (NDVI) (**e**), photochemical reflectance (PRI) (**f**), plant senescence reflectance (PSRI) (**g**), and water band (WBI) (**h**) of mustard microgreens. B0R100, B10R90, B25R75 B50R50, B75R25, and B100R0: indicate the percentage of blue (B) and red (R) light. All values in the table are expressed as mean ± standard error (*n* = 3). Means with different letters are significantly different at the *p* < 0.05 level according to Tukey’s honestly significant difference test.

Monochromatic red (B0) and blue (B100) light resulted in a significant decrease in chlorophyll and flavonol content (Figure 2). Increasing the B light percentage decreased measured index values for anthocyanin reflectance (ARI1), carotenoid reflectance (CRI2), normalized difference vegetation (NDVI), plant senescence reflectance (PSRI), and water band (WBI) and increased the value for the photochemical reflectance index (PRI) (Figure 2).

**Figure 3:**
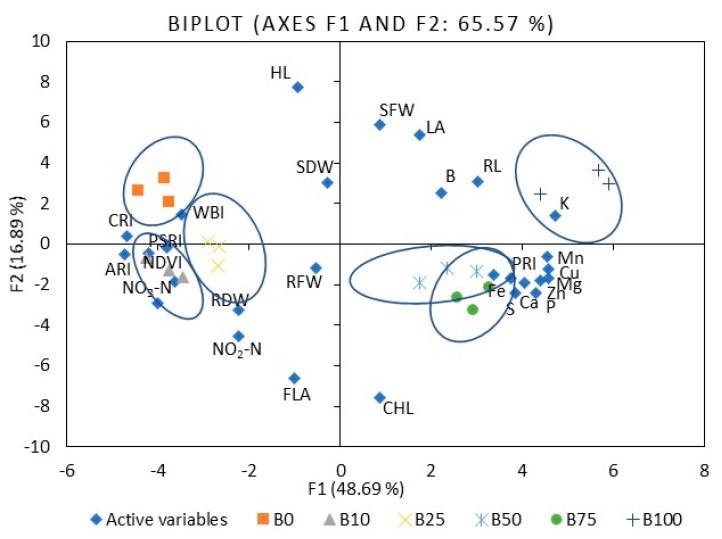
The PCA scatter plots, indicating distinct differences in the investigated parameters of mustard microgreens. B0R100, B10R90, B25R75 B50R50, B75R25, and B100R0: indicate the percentage of blue (B) and red (R) light.HL: hypocotyl length, RL: root length, LA: leaf area, SFW: shoot fresh weight, SDW: shoot dry weight, RFW: root fresh weight, RDW: root dry weight, P: phosphorus, K: potassium, Ca: calcium, Mg: magnesium, S: sulfur, Fe: iron, Zn: zinc, Mn: manganese, Cu: copper, B: boron, NO_3_-N: nitrates, NO_2_-N: nitrites, CHL: chlorophyll index, FLA: flavonoid index, ARI1: anthocyanin reflectance index, CRI2: carotenoid reflectance index, NDVI: normalized difference vegetation index, PRI: photochemical reflectance index, PSRI: plant senescence reflectance index, WBI: water band index. The significance level was set at *p* ≤ 0.05.

To compare mustard microgreen responses to different B:R light ratios, we performed PCA (Table S3). The first five principal components (F1–F5) were associated with eigenvalues of more than one and accounted for approximately 88.1% of the cumulative variability. F1, which explained 48.7% of the total variability, was mainly attributed to such mineral elements as P, K, Ca, Mg, S, Mn, Fe, Cu, and Zn. F2 accounted for 16.9% of the total variability. Factors contributing to F2 include hypocotyl and root length, leaf area, shoot fresh weight, and PSRI. F3 explained 10.3% of the variability and included N, nitrates, shoot dry weight, and root fresh and dry weight. F4 accounted for 6.8% of the population’s total variation and was mainly ascribed to B content, nitrites, ARI1, CRI2, and WBI. F5 accounted for 5.4% of the total variability and was mainly attributed to CHL, FLA, and PRI. Figure 3 illustrates the PCA of the first two components (F1 and F2). It demonstrates the relationships among the different variables (i.e., growth and nutritional quality components), where two vectors with an angle less than 90° are positively correlated. Two vectors with an angle higher than 90° are negatively correlated. For example, the mineral elements strongly positively correlated with each other, except B and N (Table S4). Mineral elements such as Mg, K, Mn, Fe, Cu, and Zn were strongly or moderately negatively correlated with ARI1, CRI2, NDVI, PSRI, and WBI. Meanwhile, PRI showed a positive, strong, or moderate correlation with the elements mentioned above. The spectral reflectance indexes were strongly or moderately positively correlated with each other, except PRI, where the correlation was negative. The F1 and F2 score plots (Figure 3) categorized treatments into six groups. The lower right quadrant shows the effect of 50% and 75% of B light and the upper effect of 100B, which differed from the treatment with a lower percentage of B light.

### 2.2. Kale

Generally, similar trends of B light exposure as those found for mustard were found for mineral nutrients in kale. However, responses depended on mineral nutrients. The highest percentage of B light (B100R0) resulted in a higher content of mostly macro and micronutrients in the kale microgreens, except Fe, of which the content was significantly higher at B75R25 compared with other lighting treatments (Table 4 and Table 5). On the other hand, high P and K content were found under B25R75, B50R50, B75R25, and B100R0. Monochromatic red light (B0R100) resulted in lower Ca and high K content (Table 4). Micronutrients, such as Zn and Cu content, under B50R50 and B75R25 were similar to those under B100R0 (Table 5). The B:R ratio had no significant effect on Mg and B content in kale.

B75R25 increased the BCF of most mineral nutrients, except Mg, of which the BCF was higher under monochromatic R light (Table S5). In addition, such light resulted in a relatively high BCF of various mineral elements compared to B10R90, B25R75, and/or B50R75 lighting.

No clear trends in TF changes were determined at different B:R ratios, which depended more on individual mineral elements (Table S6). Different B:R ratios did not affect the TF of P or Zn in kale. Monochromatic R light (B0R100) decreased the TF of Mg, S, Fe, and Cu, and B75R25 lighting decreased the TF of K, Ca, and Mn. A greater TF of Ca and Zn was found under B10R90 lighting, a greater TF of Mn was found under B25R75, and a greater TF of K, Mg, S, and Cu was found under B100R0.

**Figure 4:**
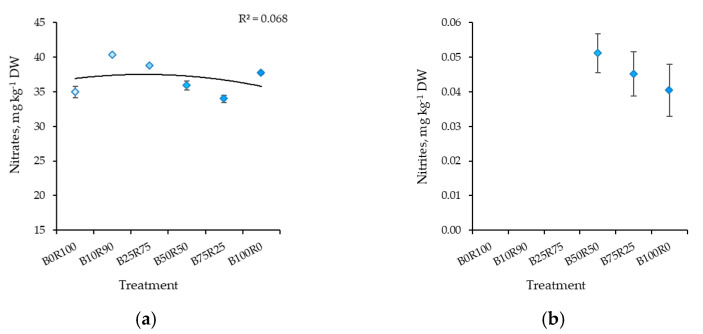
Effect of different blue–red light ratios in LED lighting on the nitrates (**a**) and nitrites (**b**) of kale microgreens. B0R100, B10R90, B25R75 B50R50, B75R25, and B100R0: indicate the percentage of blue (B) and red (R) light. All values in the table are expressed as mean ± standard error (*n* = 3). Means with different letters are significantly different at the *p* < 0.05 level according to Tukey’s honestly significant difference test.

The significantly higher content of nitrates in kale was determined under B10R90. Meanwhile, a lower content was found under B75R25, B0R100, and B50R50, respectively (Figure 4a). Nitrite levels were below the detection limit under B0R100, B10R90, and B25R75 lighting (Figure 4b). Meanwhile, other lighting treatments had no significant effect on nitrite content, but it decreased slightly as the percentage of B light increased.

Compared to mustard, the same trends of B light percentage were found in the illumination spectrum of the hypocotyl length, shoot fresh and dry weight, and shoot-to-root ratio of kale microgreens (Table 6). However, different lighting treatments did not affect the root length or leaf area. Monochromatic R (B0R100) and B (B100R0) light resulted in a significant decrease in root fresh and dry weight. These parameters were greater under B10R90, B25R75, and B50R50 lighting.

**Figure 5:**
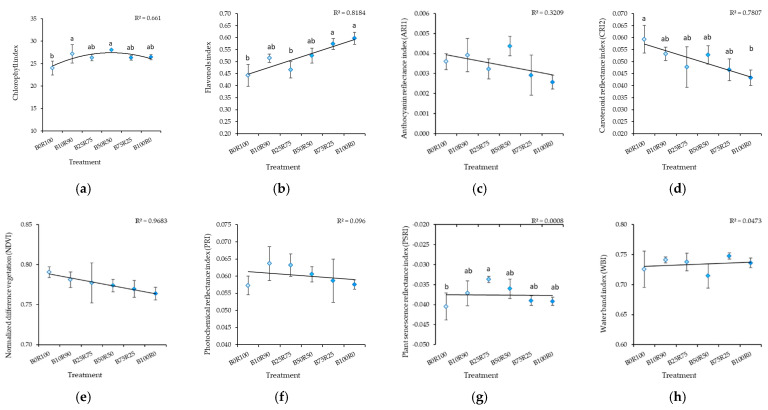
Effect of different blue–red light ratios in LED lighting on the chlorophyll (CHL) (**a**), flavonols (FLA) (**b**), anthocyanin reflectance (ARI1) (**c**), carotenoid reflectance (CRI2) (**d**), normalized difference vegetation (NDVI) (**e**), photochemical reflectance (PRI) (**f**), plant senescence reflectance (PSRI) (**g**), and water band (WBI) (**h**) indexes of kale microgreens. B0R100, B10R90, B25R75 B50R50, B75R25, and B100R0: indicate the percentage of blue (B) and red (R) light. All values in the table are expressed as mean ± standard error (*n* = 3). Means with different letters are significantly different at the *p* < 0.05 level according to Tukey’s honestly significant difference test. No difference due to ARI1, NDVI, PRI, or WBI was found.

Monochromatic R light (B0R100) resulted in a lower CHL, FLA, and PSRI in kale microgreens in comparison with B light treatments (Figure 5a,b,g). Increasing the B:R ratio increased FLA and decreased CRI2 (Figure 5b,d). Different B:R ratios did not affect ARI1 (Figure 5c), NDVI (Figure 5e), PRI (Figure 5f), or WBI (Figure 5h).

**Figure 6:**
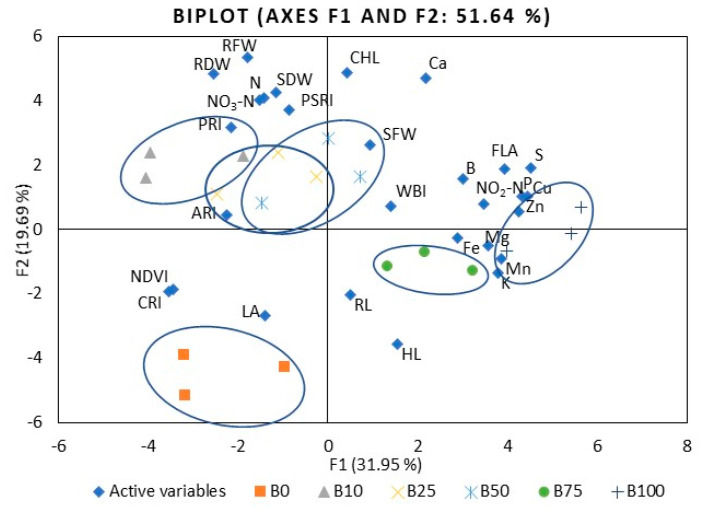
The PCA scatter plots, indicating distinct differences in investigated parameters of kale microgreens. B0R100, B10R90, B25R75 B50R50, B75R25, and B100R0: indicate the percentage of blue (B) and red (R) light. HL: hypocotyl length, RL: root length, LA: leaf area, SFW: shoot fresh weight, SDW: shoot dry weight, RFW: root fresh weight, RDW: root dry weight, P: phosphorus, K: potassium, Ca: calcium, Mg: magnesium, S: sulfur, Fe: iron, Zn: zinc, Mn: manganese, Cu: copper, B: boron, NO_3_-N: nitrates, NO_2_-N: nitrites, CHL: chlorophyll index, FLA: flavonoid index, ARI1: anthocyanin reflectance index, CRI2: carotenoid reflectance index, NDVI: normalized difference vegetation index, PRI: photochemical reflectance index, PSRI: plant senescence reflectance index, WBI: water band index. The significance level was set at *p* ≤ 0.05.

The first six principal components (F1–F6) of kale microgreens were associated with eigenvalues of more than 1 and accounted for approximately 85.7% of the cumulative variability (Table S7). F1, which explained 31.9% of the total variability, was mainly attributed to such mineral elements as P, K, Mg, S, Fe, Cu, and Zn, nitrites, and FLA. F2 accounted for 19.7% of the total variability. Factors contributing to F2 include the shoot dry weight, root fresh and dry weight, CHL, and PSRI. F3 explained 13.4% of the variability, including Mn content, hypocotyl and root length, leaf area, shoot fresh weight, and NDVI. F4 accounted for 8.3% of the total variation in the population and was mainly ascribed to WBI. F5 accounted for 6.8% of the total variability and was mainly attributed to PSRI. F6 explained 5.5% of the variability; this included factors such as B content, ARI1, and CRI2. Figure 6 illustrates the PCA of the first two components (F1 and F2) and demonstrates the relationships among the kale microgreens’ different variables. Kale showed fewer correlations between different indexes compared to mustard. For example, S and P were strongly or moderately positively correlated with most other mineral elements (Table S8), where Mn was correlated with Mg, K, Cu, and Zn, Zn with Fe and Cu, K with Mg, and the flavonol index with Fe, Cu, Zn, B, P, S, and nitrites. Mineral elements such as Mg, Ca, Cu, Zn, B, P, and S were moderately negatively correlated with CRI2 and NDVI. The F1 and F2 score plots (Figure 6) categorized treatments into six groups. The right quadrant mainly shows the effect of B75R25 and B100R0. The lower left quadrant shows the effect of monochromatic R light (B0), which differed from the other treatments.

## 3. Discussion

### 3.1. Mineral Nutrients

Literature data have reported that Brassicaceae microgreens are an excellent source of macro- and micronutrients in the human diet. Various studies have reported that mineral nutrient content is closely related to microgreen species and varieties and their maturity stages, cultivation seasons, and different environmental factors during growth, including light [5,15]. Our results showed that, generally, a higher percentage of blue (B50R50–B100R0) light in the illumination spectrum resulted in a higher content of mineral nutrients in the mustard and kale microgreens. According to the literature, blue light through the control of the blue light receptor phototropin (Phot 1 and Phot 2) causes an opening of ion channels located on cell plasma membranes and promotes the flux of ion transport [21,35,36,37]. Some studies have confirmed the positive effect of blue light on the accumulation of mineral nutrients in various microgreens [21,38,39,40]. Moreover, other studies have reported a positive red light effect on mineral nutrient content in buckwheat and beet microgreens [28,38]. These studies have mainly investigated short-term blue light as supplemental to other illumination or the long-term effects of monochromatic blue and red light or their dichromatic illumination with one ratio.

We have not found data on the impact of the wide range of dichromatic blue and red lights on mineral nutrient content in vegetable microgreens. However, Bartucca and co-authors [41] studied blue–red ratios from B0 to B100, revealing similar mineral nutrient content trends in einkorn seedlings. Meanwhile, there were no differences in mineral nutrient content in *Brassica* microgreens when comparing R80B20 and R20B80 [42]. Higher mineral nutrient content was determined in mustard under 25% of blue light and in red pak choi and tatsoi under 33% of blue light when 0%, 8%, 16%, 25%, and 33% of blue light in combination with red or far-red light were studied [38].

Various studies have shown the increasing popularity of microgreens and revealed information about their bioactive compounds and response to LED lighting. However, there is little published data on mineral nutrients in microgreens, and discussions of their changes depending on different levels of light are based on data on other vegetables [15,17]. Data on the effect of monochromatic blue, red, and dichromatic blue–red light of different proportions on mineral nutrient content in various vegetables are contradictory and depend on genotype and individual mineral nutrients. For example, some authors have reported that the mineral nutrient content in marigold was not significantly different among treatments with various percentages of blue light [30]. Combinations with a more significant percentage of red light resulted in increases in P, K, Ca, and Zn in dill [43], in N, P, K, and Mg in lettuce [23,31], and in various mineral nutrients in basil [24,44]. Meanwhile, a higher percentage of blue light resulted in increases in S, Mg, and B in basil [44] and in Ca in lettuce [31]. The current study also showed that genotype or individual mineral nutrients content depends on the response to different blue–red light compositions. For example, both microgreens had a high content of P, Ca, Mg and B under lighting with higher blue light proportions (B50R50, B75R25, and B100R0), but a higher S content was found only in mustard and higher K, Zn, and Cu was found only in kale. Meanwhile, B75R25 resulted in a higher content of all micronutrients except for Mn in kale.

On the other hand, many of the studies reviewed above showed a positive effect of red light on the mineral nutrient content in various plants. Some studies with *Arabidopsis* revealed that red light, through phytochrome photoreceptors, should also be involved in mineral nutrient uptake and stimulation in roots via multiple routes [45]. However, in our study, the results showed that red light had no such effect on mineral nutrient concentrations in microgreens.

Various studies have demonstrated how different blue and red LED light ratios affect the mineral nutrient uptake in shoots, but there is a lack of data regarding the effect on mineral nutrient uptake to roots from a hydroponic nutrient solution and from roots to shoots. In order to estimate this, we calculated the bioconcentration (BCF) and translocation (TF) factors. The BCF represents the plant’s effectiveness in accumulating the element compared to its concentration in soils or solutions. By contrast, the TF indicates the plant’s efficiency in transferring nutrients from roots to shoots. If the value for either of these factors is higher than 1, then the plants can be accumulators; equal to 1 means no influences; less than 1 means the plant can be an excluder. Calculations of BCF and TF are mostly related to the assessment of heavy metals or uptake of one or several trace elements from soil or hydroponic solution [46,47,48,49]. We applied this to the assessment of mineral nutrient uptake in microgreens. Our study showed that the BCF was higher than 1, meaning that the accumulation of mineral nutrients from a solution to roots was effective in both microgreens, which could be caused by an early growth phase and rapid growth.

Literature data have shown that younger plants are better able to absorb metal ions, and it is assumed that this might be related to more intense transpiration during leaf expansion and stomata development [50]. However, our study revealed the species-dependent response of BCF and TF to different blue–red light ratios. In mustard, higher BCF was determined under B25R75 and B50R50 lighting, but in kale, it was under B75R25, except in the case of BCF_Mg_, which had a higher value under B0R100. Meanwhile, the TF values of P, K, Fe, Zn, and Cu in both microgreens were lower than 1.

According to the literature, a minimal metal transfer to shoots might be possible due to metal sequestration in plant root vacuoles, where metals are fixed as nontoxic elements [48]. Our study showed that the blue–red light ratio affected mineral nutrient translocation in microgreens differently. In both microgreens, a higher TF was determined under lighting with a higher proportion of blue light (B50R50–B100R0). However, TF values for P, K, Mg, and S were high under monochromatic red light in mustard. Meanwhile, different lighting did not affect the TF values of P and Zn in kale.

In summary, our study shows that species and mineral nutrients content respond differently to different blue–red light compositions. This suggests several pathways in which light regulates nutrient uptake in roots, even though it cannot penetrate more than a few millimeters into the soil [45,51]. Xu and co-authors [37] reviewed data from various studies and proposed a model of multiple light signaling pathways regulating nutrient uptake. According to them, light signaling and photosynthesis modulate pathways of [Ca_2_^+^] cyt concentrations, hormones, LONG HYPOCOTYL 5 (HY5), sugar, and microRNA, which regulate Fe, Cd, Cu, S, and NO_3_^−^ ion uptake and use through various mechanisms. Phytochrome B (PHYB) is also expressed in roots and affects P uptake. On the other hand, studies analyzing the effect of lighting on mineral nutrient uptake in plants mostly focus on monochromatic blue or red lighting, but data in the literature have shown that a mixed light spectrum of red and blue light is more effective for improving nutrient uptake. However, the mechanism of nutrient absorption regulated by such light remains to be clarified in horticultural crops [45,51], especially in such specialty crops as microgreens.

### 3.2. Nitrates and Nitrites

Red light can efficiently reduce the nitrate concentration in plants by stimulating the activity of NR through phytochromes [52]. Short-term 638 nm or monochromatic 660 nm red light resulted in decreased nitrate content in various leafy vegetables, including Brassicaceae [14,53], which are characterized by a high nitrate accumulation capacity [54]. However, we determined significantly lower levels of nitrates in both Brassicaceae microgreens according to the last amendment of the European Commission Regulation limits. This document indicates that the nitrate concentration in leafy vegetables depending on the season of cultivation must be up to 3500 mg kg^–1^ FW (fresh weight) for spinach, 4000–5000 mg kg^–1^ FW for lettuce, grown under cover, and 6000–7000 mg kg^–1^ FW for rucola [55].

We revealed a reduction in nitrates in kale microgreens under monochromatic red light, but this reduction was similar to that under lighting with a higher percentage of blue light (B50R50 and B75B25). Some studies have also revealed that the combination of red and blue light was more effective for decreasing nitrate content in different plants [13,54,56]. However, there is a lack of information about the impact of different blue–red light ratios. Relatively low nitrate content was found in mustard, red pak choi, and tatsoi microgreens under blue, red, and far-red LED lighting with 25% blue light (455 nm) when blue light of 0%, 8%, 16%, 25%, and 33% was investigated [40]. Other studies have suggested that the best red–blue light ratio to reduce nitrate levels in hydroponic lettuce is 8:1 or 4:1 [13]. This study showed that a higher percentage of blue light in blue–red lighting led to a higher reduction of nitrates in microgreens. Meanwhile, other studies have revealed that nitrate levels in microgreens and lettuce were unaffected by LEDs when varying blue light proportion [57,58,59].

One of the metabolic stages in nitrate assimilation is its reduction to nitrite initiated by NR [57]. Though clinical studies do not clearly confirm this, it has been stated that nitrates in the human body can be transformed into nitrites, which react with amines and amides to produce N-nitroso compounds that might increase the risk of certain forms of cancer [13]. According to the literature, nitrite levels in various leafy vegetables ranged between 1.1 and 57 mg kg^−1^ FW, and the tolerance limit is 4 mg kg^−1^ FW [60]. According to our results, nitrite content was less this limit in both microgreens’ species.

However, nitrite content is rarely considered when assessing the effect of lighting on changes in vegetable quality. Our study revealed that a higher percentage of blue light (B75R25 and B100R0) had a positive effect on nitrite reduction in mustard microgreens. On the other hand, monochromatic red light had a similar effect. Red light is more effective in N use, but additional research has suggested that blue light was also involved in this process. The molecular mechanisms of this process are not fully understood but may include phytochromes, which can weakly absorb blue light in addition to red and far-red light [47]. This suggests that under lighting with a higher proportion of blue light, phytochromes can absorb more blue light and more effectively enhance NR and NiR activity. However, Bian and co-authors [61] reported that nitrite content in plants was similar among different light spectral treatments. Meanwhile, in other studies, nitrite levels have been detected in more vegetable species in wintertime than in summer. This was explained by the fact that NiR activity is related to the function of photosystem I and highly dependent on light. Under lower light levels in winter, NiR activity is suppressed, and nitrite accumulation increases [54].

It should be noted that the nitrite content in kale microgreens was very low compared to that in mustard, though the nitrate content was similar in both microgreens. Nitrite content was not detected under B0R100 nor B25R75 lighting, and B50R50 and B100R0 lighting had no significant effect. Other studies have also reported genotypic variation in nitrite content in various vegetables [54].

### 3.3. Growth

Hypocotyls are one of the main edible parts of microgreens. Longer hypocotyls are more attractive to most producers since they are easy to harvest. Therefore, their length is an essential quality attribute and may be affected by blue–red light [15,62]. The blue light-sensing cryptochrome (cry) and the red/far-red light-sensing phytochrome (phy) may regulate hypocotyl length due to the activation of COP1 (CONSTITUTIVE PHOTOMORPHOGENIC 1), a repressor for photomorphogenesis, and the stabilization of HY5 (LONGHYPOCOTYL 5) and HYH (LONG HYPOCOTYL 5 HOMOLOG), transcription factors that promote photomorphogenesis [18,63,64]. Our study revealed that monochromatic blue and red light similarly promoted hypocotyl elongation of both investigated microgreens. This is consistent with other studies [18,64,65]. However, blue–red light, compared with monochromatic blue or red light, resulted in shorter hypocotyls. We observed a trend that the length of hypocotyls decreased with an increasing proportion of blue light, but there were only significantly shorter hypocotyls in the mustard microgreens under B75 light. Kong and co-authors [64] hypothesized that the blue light effect on elongation is related to phytochrome activity, at least in some cases. A stronger inhibitory effect of blue–red light, compared with monochromatic red or blue light, has been found in studies with other plants, such as various bedding plants [64], buckwheat sprouts [18], and Brassicaceae microgreens [66]. The higher proportion of blue light in blue–red lighting has had a stronger inhibitory effect on hypocotyl elongation [44,65,66,67].

Our study showed the different responses of microgreen growth parameters to blue–red light and revealed species differences. Monochromatic blue light resulted in increased shoot fresh weight in both microgreens, though a higher blue light percentage in blue–red lighting tended to result in a decrease. However, a different blue–red ratio did not affect the shoot-to-root ratio, which significantly increased under B100R0 in mustard and under B0R100 and B100R0 in kale, mostly due to a lower root dry weight. Lighting treatments did not affect root fresh or dry weight in mustard nor root length or leaf area in kale. A species-dependent response to blue–red lighting has also been found in other studies. For example, various microgreen species showed no differences in the fresh yield under blue light percentage variation within the tested range [26,60,66]. However, blue light enhanced the fresh mass of basil microgreens [68]. Other authors have supposed that increasing the proportion of blue light reduces the fresh and dry mass of plants, such as cucumber [65], basil [24,45], and lettuce [20,50].

Some differences in the results between microgreens and other plants might be due to different growth stages and agrotechnical features. Most microgreens are harvested at the growth stage of two fully expanded cotyledons and one real leaf, i.e., as they transition from heterotrophic growth, which depends on the nutrients stored in the seeds to autotrophic growth, which depends on photosynthetic assimilates. Therefore, microgreens, rather than other plants, might be affected by light treatments that are not long enough, causing a significantly different photosynthesis response [64].

In addition, microgreens grow very densely, and competition between them can result in different responses to lighting compared to other plants. Changes in light conditions caused by the proximity of competitors provoke shade avoidance responses, such as stem elongation and leaf hyponasty, allowing the plant to outgrow others and increase competitive performance [69].

### 3.4. Leaf Reflectance As an Indicator of Nutritional Value Assessment

Numerous studies have shown that red, blue, or red-blue LED light can affect the accumulation of bioactive compounds, flavor, and pigmentation of microgreens. However, these studies often show differing results of such light on various phytochemicals, varying from positive to adverse effects and, sometimes, with no effect being observed. In most cases, the impact of monochromatic blue light, red light, and a single B:R ratio is studied, with few studies examining different B:R ratios [17,18,39]. Kamal et al. [42] revealed no difference between R80:B20 and R20:B80 lighting on the α-tocopherol, ascorbic acid, and β-carotene phylloquinone contents in Brassicaceae microgreens. The total carotenoid concentration was unaffected by the increased blue light percentage in various Brassicaceae, basil, and parsley microgreens [26,35,68,70]. The greatest quantity of total anthocyanins and flavonols was found in tatsoi under 25% and in basil under 16–33% of blue light [67]. Phenolic synthesis and free radical scavenging activity were improved by predominantly red light for green basil and by predominantly blue light for red basil [69]. In the abovementioned studies, the contents of bioactive compounds were determined using spectrophotometric and/or chromatographic methods. In the present study, we assessed changes in microgreen quality using nondestructive leaf reflectance measurement methods that rapidly estimate numerous functionally significant leaf parameters [71].

Our results show the contrasting genotypic responses of microgreens to blue and red light, which have also been demonstrated in other studies [35,55,68,70]. An increased blue light percentage resulted in an increased flavonol index (FLA) value and a decreased carotenoid reflectance index (CRI2) value in kale microgreens. Values for other leaf reflectance indexes were practically unaffected by B:R lighting treatments. Mustard microgreens were more flexible in terms of the blue–red light ratio. An increasing blue–red light ratio decreased the values of such indexes as ARI1, CRI2, NDVI, and PSRI and increased PRI, which was strongly or moderately negatively correlated with indexes mentioned earlier. Meanwhile, monochromatic red (B0R100) and blue (B100R0) light resulted in a decrease in chlorophyll (CHL) and flavonol (FLA) contents and variations in the B:R ratios did not affect these indexes. Contrary trends were noticed for CRI2, which indicates a greater carotenoid concentration relative to chlorophyll and PRI, which is indicative of changes in the xanthophyll cycle. Other studies have also shown different B:R ratio effects depending on the type of carotenoid [26,70]. On the other hand, a PRI increase could indicate greater photosynthetic efficiency due to a relatively high chlorophyll content, which can be deduced from the lower values of ARI1, CRI2, and PSRI [72]. This could suggest that a higher proportion of blue light resulted in greater photosynthetic efficiency [73]. Meanwhile, PRI showed a strong or moderate positive correlation with most mineral nutrients.

## 4. Materials and Methods

### 4.1. Plant Materials and Growth Conditions

The experiments were performed in closed, controlled, walk-in growth chambers (4 m × 6 m) in the phytotron complex at the Institute of Horticulture (IH), Research Centre for Agriculture and Forestry, Lithuania. The microclimate in the growth chamber was autonomously and independently controlled using the Phytotron Microclimate Control System developed in IH based on separate microcontrollers (AL-2-24MR-D, Mitsubishi Electric, Tokyo, Japan). The air temperature was measured with resistance temperature detectors (P-100; OMEGA Engineering Ltd., Norwalk, CT, USA), and data for these measurements were transmitted to the microcontrollers. The relative humidity and CO_2_ concentration were measured by capacitive sensors (CO_2_RT(-D); Regin, Kållered, Sweden) and controlled by additional humidifiers. Data were collected every minute, processed, and stored on the operator panel (E1000, Mitsubishi Electric, Tokyo, Japan).

Two different genotypes of microgreens were used in the experiments: mustard (*Brassica juncea* L. ‘Red Lace’) and kale (*Brassica oleracea* L. ‘Red Russian’). Respectively, 2.5–5 g of seeds (CN Seeds, Cambridgeshire, UK) were sown on the surface of the hydroponic seed sprouting tray (0.8 m^2^) with perforated paper and deionized water (pH: 5.5–5.6) and covered with a plastic lid. After four days, trays were uncovered, and deionized water was exchanged with modified Hoagland nutrient solution containing the following average nutrient concentrations [mg L^−1^]: N, 120; P, 20; K, 128; Ca, 72; Mg, 40; S, 53; Fe, 4; Mn, 0.08; Cu, 0.08; B, 0.16; Zn, 0.8. The pH was 5.5–6.5, and the electrical conductivity (EC) was 1.3–1.7 mS cm^−1^ (GroLine HI9814, Hanna Instruments, Woonsocket, RI, USA). One hydroponic tray represented one replicate. Two trays were used under each radiation condition. The experiments were repeated twice. Seeds were germinated over 18 h with day/night temperatures (± SD) of 21/17 ± 2 °C and a relative air humidity of 60% ± 5%.

### 4.2. Lighting Conditions

Microgreens were cultivated under a controllable lighting fixture (HLRD, Hortiled, Kaunas, Lithuania) consisting of blue (447 nm) and red (660 nm) light-emitting diodes (LEDs). The total illuminated area for each treatment was 0.4 m^2^. In six treatments, red and blue LEDs were used at different PFD ratios: 0%B:100%R, 10%B:90%R, 25%B:75%R, 50%B:50%R, 75%B:25%R and 100%B:0%R (treatments code B0R100, B10R90, B25R75, B50R50, B75R25, and B100R0, respectively). All lighting treatments delivered the same total photon flux density (TPFD) of 250 μmol m^−2^ s^−1^ (Table 7). The photon distributions of all lighting treatments were measured using a portable photometer–radiometer at the tray surface level (RF-100, Sonopan, Bialystok, Poland).

### 4.3. Sampling and Measurements

Microgreen cotyledons were harvested with stems near the ground level. Samples were harvested from the center of the container, leaving plants in the 1.5 cm edge as a guard. The dry and fresh weight of microgreens was determined by the gravimetric method using an electronic analytical balance (Mettler Toledo AG64, Columbus, OH, USA) and was used to calculate the shoot/root ratio. The leaf area of microgreens was measured using the WinDIAS meter (Delta-T Devices Ltd., Cambridge, UK). Biometric measurements and fresh and dry weights were performed on thirty plants and nondestructive measurements on ten plants of three replications that were randomly selected from the edges and middle of each tray. Samples of microgreens used for mineral nutrients, nitrates and nitrites composition analysis as well as for dry weight were washed with deionized water and dried at 70 °C for 48 h in a drying oven (Venticell 222, MBT, Brno-Zábrdovice, Czech Republic). Conjugated ex-periment samples for mineral nutrients, nitrate and nitrite determination were stored in tightly closed 50 mL plastic bags until analysis. They analysis was performed in 3 biological and 3 analytical replications.

### 4.4. Nondestructive Measurements

Nondestructive measurements of leaf chlorophyll (CHL) and flavonol (FLA) indexes were performed using the Dualex 4 Scientific^®^ (FORCE-A, Orsay, France) meter.

Spectral reflectance was measured using a leaf spectrometer (CID Bio-Science, Camas, WA, USA) from 9 to 12 a.m. Reflection spectra obtained from the leaves were used to calculate various indexes according to formulas presented by produces. The anthocyanin reflectance index (ARI1), which shows changes in the anthocyanin content:

ARI1 = 1/R550 − 1/R700(1)

The carotenoid reflectance index (CRI2) shows changes in the carotenoid-to-chlorophyll ratio:

CRI2 = 1/R510 − 1/R700(2)

The normalized difference vegetation index (NDVI) shows changes in biomass content:

NDVI = (R800 − R680)/(R800 + R680)(3)

The photochemical reflectance index (PRI) shows changes in the xanthophyll cycle:

PRI = (R531 − R570)/(R531 + R570)(4)

The plant senescence reflectance index (PSRI) shows changes in dry or senescent carbon:

PSRI = (R680 − R500)/R750(5)

The water band index (WBI) shows changes in leaf water content:

WBI = R900/R970(6)

R970, R900, R800, R750, R700, R680, R570, R550, R531, R510, and R500 represent the leaf reflectance integrated over a 10 nm wavelength band centered on 970, 900, 800, 750, 700, 680, 570, 550, 531, 510, and 500 nm, respectively.

### 4.5. Determination of Mineral Nutrients and Their Bioconcentration and Translocation Factors

The contents of macronutrients (K, Ca, Mg, S, and P) and micronutrients (Fe, Zn, and Mn) in microgreens were determined using a modified microwave-assisted digestion technique combined with ICP–OES methods as described by Araújo et al. [74] and Barbosa et al. [75]. The complete digestion of 0.5 g of powdered shoot and 0.1 g of root material was achieved with 8 mL of 65% HNO_3_ using a microwave-assisted digestion system (Multiwave GO; Anton Paar GmbH, Graz, Austria), following a two-step heating program: (1) heating to 150 °C in 3 min and held for 10 min, and (2) heating to 180 °C in 10 min and held for 10 min, followed by cooling. The mineralized samples were diluted to 50 mL with ultrapure deionized water, filtered with Whatman Grade 1 qualitative filter paper, and stored at 4 °C until analysis. The nutrient profile was analyzed by ICP–OES (SPECTRO Genesis spectrometer, Analytical Instruments GmbH, Kleve, Germany). The contents of mineral nutrients (mg L^−1^) were evaluated according to analytical wavebands of 766.491 nm for K, 445.478 nm for Ca, 279.079 nm for Mg, 257.611 nm for Mn, 259.941 nm for Fe, 213.856 nm for Zn, 213.618 nm for P, and 182.034 nm for S. The following plasma conditions were adopted: 1.3 kW RF power, 1.0 L min^−1^ auxiliary argon (Ar) flow, 0.50 L min^−1^ nebulizer Ar flow, 12 L min^−1^ coolant Ar flow, and axial plasma configuration. Each sample was analyzed in triplicate. The calibration standards of mineral nutrients were prepared by diluting an ICP multielement standard solution (1000 mg L^−1^) with 6.5% HNO_3_ and by diluting phosphorus and standard sulfur solutions (1000 mg L^−1^) with ultrapure deionized water (Merck KGaA). A separate calibration curve was employed for each mineral nutrient using the blank 6.5% HNO_3_ solution (although ultrapure deionized water was employed for S and P) as a zero point, and calibration solutions were prepared at concentrations of 0.01–10 mg L^−1^ for Zn, Fe and Mn and 1.0–400 mg L^−1^ for K, Ca, Mg, P and S. The content of each mineral nutrient was recounted as mg g^−1^ dry weight.

The mineral nutrient bioconcentration factor (BCF) of the roots and shoots was calculated as follows:

BCF = mineral nutrient concentration in the plant material (mg kg^−1^)/mineral element concentration in the solution (mg kg^−1^) (7)

The translocation factor (TF) was calculated to evaluate the plant’s ability to accumulate the mineral elements, absorbed by roots, in the aerial part:

TF = mineral nutrient concentration in the shoot (mg kg^−1^)/mineral element concentration in the roots (mg kg^−1^) (8)

The BCF and TF formulas were modified according to the formulas for calculating such factors for various heavy metals and trace elements [48,49,50].

### 4.6. Determination of Nitrate and Nitrite

The nitrate and nitrite contents were determined using the spectrophotometric method, as described in detail by Merino [76]. Plant material was oven-dried at 70 °C for 48 h. Samples were prepared by hot water (70 °C; 1:100, w/v) extraction from dry plant material in an ultrasonic bath for 30 min and clarified using Carrez solution. In each sample, initial nitrite concentration and total nitrite after nitrate reduction to NO_2_ in the presence of zinc powder were determined by diazotizing with sulfanilamide and coupling with N-(1-naphthyl)-ethylenediamine dihydrochloride to form a highly colored azo dye that was measured at 540 nm (M501, Camspec, Leeds, UK). The nitrite present in the sample was analyzed without the reduction step. The nitrate was calculated as the difference between the total nitrite content after reduction and the initial nitrite concentration. Nitrate and nitrite amounts (mg kg^−1^) were deduced from a calibration curve and expressed on the basis of dry plant weight.

### 4.7. Statistical Analysis

Statistical analysis was performed using Microsoft Excel 2016 and Addinsoft XLSTAT 2019.1 XLSTAT statistical and data analysis (Long Island, NY, USA). The data are presented as means of three replicates (*n* = 3) linked to the sampling points. One-way analysis of variance (ANOVA) followed by Tukey’s honestly significant difference test (*p* < 0.05) for multiple comparisons was used to evaluate differences between means of measurements. The principal component analysis (PCA) was performed at a 99% significance level. The results presented in PCA scatter plots indicate distinct effects of lighting treatments on the levels of minerals and correlation circles (based on Pearson’s correlation matrix), which summarize the relationships between the investigated indexes in microgreens under the light treatments.

## 5. Conclusions

In summary, the present study’s results demonstrate that a higher percentage of blue (B75R25 and B100R0) light positively affected the accumulation of mineral nutrients in Brassicaceae microgreens and that such conditions could be strategically used as a tool for biofortification. However, such light resulted in lowers yield and shorter hypocotyls. Meanwhile, our results indicated species-dependent responses; mustard microgreens were more flexible in terms of the effect of the blue–red light ratio concerning decreased nutritional value, contrary to the absence of any impact on kale. In addition, our results show that technological and nutritional quality should not always be combined. In this case, 25% or 50% of blue light is recommended because, at this range, microgreens had sufficiently high yields and levels of mineral nutrients.

## Figures and Tables

**Table 1:** Effect of different blue–red light ratios in LED lighting on the macronutrients of mustard microgreens, mg g^−1^ DW.

Treatment	Phosphorus	Potassium	Calcium	Magnesium	Sulfur
B0R100	8.27 ± 0.12 b	22.44 ± 0.43 c	16.92 ± 0.12 c	5.21 ± 0.07 b	13.78 ± 0.26 bc
B10R90	8.04 ± 0.14 b	22.62 ± 0.17 c	16.43 ± 0.13 c	5.24 ± 0.07 b	13.62 ± 0.17 c
B25R75	8.40 ± 0.20 b	22.61 ± 0.16 c	16.78 ± 0.28 c	5.36 ± 0.11 b	13.44 ± 0.10 c
B50R50	9.84 ± 0.10 a	24.37 ± 0.11 b	19.01 ± 0.25 a	5.90 ± 0.10 a	14.66 ± 0.14 a
B75R25	9.78 ± 0.17 a	24.67 ± 0.20 b	18.37 ± 0.18 ab	5.86 ± 0.11 a	14.77 ± 0.14 a
B100R0	9.34 ± 0.38 a	26.53 ± 0.54 a	17.82 ± 0.51 b	5.86 ± 0.19 a	14.39 ± 0.46 ab

B0R100, B10R90, B25R75 B50R50, B75R25, and B100R0: indicate the percentage of blue (B) and red (R) light; DW: dry weight. All values in the table are expressed as mean ± standard error (*n* = 3). Means with different letters are significantly different at the *p* < 0.05 level according to Tukey’s honestly significant difference test.

**Table 2:** Effect of different blue–red light ratios in LED lighting on the micronutrients of mustard microgreens, mg g^−1^ DW.

Treatment	Manganese	Iron	Zinc	Copper	Boron
B0R100	0.062 ± 0.000 bc	0.094 ± 0.002 d	0.078 ± 0.001 de	0.0099 ± 0.0004 d	0.029 ± 0.001 ab
B10R90	0.060 ± 0.001 c	0.114 ± 0.002 b	0.080 ± 0.000 d	0.0103 ± 0.0001 b	0.029 ± 0.002 ab
B25R75	0.065 ± 0.002 b	0.100 ± 0.002 cd	0.076 ± 0.001 e	0.0102 ± 0.0001 cd	0.025 ± 0.002 b
B50R50	0.068 ± 0.001 a	0.102 ± 0.001 c	0.089 ± 0.001 c	0.0112 ± 0.0002 c	0.034 ± 0.006 a
B75R25	0.070 ± 0.001 a	0.129 ± 0.003 a	0.097 ± 0.001 a	0.0122 ± 0.0002 a	0.026 ± 0.001 ab
B100R0	0.070 ± 0.002 a	0.126 ± 0.003 a	0.093 ± 0.003 b	0.0121 ± 0.0002 a	0.033 ± 0.001 a

B0R100, B10R90, B25R75 B50R50, B75R25, and B100R0: indicate the percentage of blue (B) and red (R) light; DW: dry weight. All values in the table are expressed as mean ± standard error (*n* = 3). Means with different letters are significantly different at the *p* < 0.05 level according to Tukey’s honestly significant difference test.

**Table 3:** Effect of different blue–red light ratios in LED lighting on the growth parameters of mustard microgreens.

Treatment	Hypocotyl Length, cm	Root Length, cm	Leaf Area, cm^2^	Shoot Fresh Weight, mg	Shoot Dry Weight, mg	Root Fresh Weight, mg	Root Dry Weight, mg	Shoot-to-Root Ratio
B0R100	3.37 ± 0.10 a	6.95 ± 0.42 ab	2.43 ± 0.31 b	68.65 ± 5.07 ab	4.10 ± 0.86 a	10.82 ± 1.04 a	0.68 ± 0.03 a	5.95 ± 0.98 ab
B10R90	2.49 ± 0.14 b	6.30 ± 0.13 b	2.19 ± 0.19 b	63.20 ± 4.05 ab	4.14 ± 0.09 a	11.97 ± 0.09 a	0.75 ± 0.04 a	5.51 ± 0.16 b
B25R75	2.30 ± 0.26 b	6.53 ± 0.27 ab	2.73 ± 0.19 a	70.46 ± 4.23 ab	4.27 ± 0.48 a	12.46 ± 2.64 a	0.78 ± 0.04 a	5.46 ± 0.44 b
B50R50	2.15 ± 0.14 b	6.80 ± 0.55 ab	2.30 ± 0.15 b	60.07 ± 1.54 b	3.60 ± 0.35 b	11.60 ± 2.31 a	0.69 ± 0.09 a	5.24 ± 0.25 b
B75R25	1.38 ± 0.20 c	7.14 ± 0.23 ab	2.25 ± 0.11 b	60.99 ± 3.52 b	3.79 ± 0.24 b	11.25 ± 0.50 a	0.70 ± 0.06 a	5.43 ± 0.23 b
B100R0	3.29 ± 0.22 a	7.46 ± 0.42 a	3.08 ± 0.23 a	78.73 ± 13.21 a	4.41 ± 0.77 a	11.34 ± 1.51 a	0.62 ± 0.13 a	7.17 ± 0.79 a

B0R100, B10R90, B25R75 B50R50, B75R25, and B100R0: indicate the percentage of blue (B) and red (R) light. All values in the table are expressed as mean ± standard error (*n* = 3). Means with different letters are significantly different at the *p* < 0.05 level according to Tukey’s honestly significant difference test.

**Table 4:** Effect of different blue–red light ratios in LED lighting on the macronutrients of kale microgreens, mg g^−1^ DW.

Treatment	Phosphorus	Potassium	Calcium	Magnesium	Sulfur
B0R100	7.89 ± 0.17 bc	23.56 ± 0.36 ab	14.71 ± 0.12 b	5.67 ± 0.05 a	10.3 ± 0.45 e
B10R90	7.70 ± 0.26 bc	22.53 ± 0.64 b	15.53 ± 0.47 ab	5.57 ± 0.22 a	10.90 ± 0.06 d
B25R75	8.56 ± 0.08 a	23.51 ± 0.26 ab	16.33 ± 0.09 a	5.71 ± 0.08 a	12.30 ± 0.20 c
B50R50	8.39 ± 0.28 ab	23.30 ± 0.27 ab	16.34 ± 0.41 a	5.61 ± 0.15 a	13.19 ± 0.23 b
B75R25	8.51 ± 0.26 a	23.26 ± 0.43 ab	15.48 ± 0.39 ab	5.70 ± 0.14 a	13.86 ± 0.30 b
B100R0	8.90 ± 0.17 a	24.46 ± 0.55 a	16.20 ± 0.37 a	5.88 ± 0.14 a	14.95 ± 0.19 a

B0R100, B10R90, B25R75, B50R50, B75R25, and B100R0: indicate the percentage of blue (B) and red (R) light; DW: dry weight. All values in the table are expressed as mean ± standard error (*n* = 3). Means with different letters are significantly different at the *p* < 0.05 level according to Tukey’s honestly significant difference test.

**Table 5:** Effect of different blue–red light ratios in LED lighting on the micronutrients of kale microgreens, mg g^−1^ DW.

Treatment	Manganese	Iron	Zinc	Copper	Boron
B0R100	0.056 ± 0.000 b	0.082 ± 0.001 c	0.051 ± 0.001 b	0.0052 ± 0.0001 b	0.018 ± 0.001 a
B10R90	0.054 ± 0.001 c	0.090 ± 0.003 bc	0.052 ± 0.002 b	0.0056 ± 0.0002 b	0.022 ± 0.005 a
B25R75	0.056 ± 0.000 b	0.083 ± 0.001 c	0.053 ± 0.001 b	0.0059 ± 0.0001 b	0.018 ± 0.003 a
B50R50	0.055 ± 0.001 bc	0.084 ± 0.004 c	0.055 ± 0.005 ab	0.0076 ± 0.0001 a	0.021 ± 0.002 a
B75R25	0.055 ± 0.001 bc	0.103 ± 0.004 a	0.060 ± 0.001 a	0.0079 ± 0.0011 a	0.021 ± 0.006 a
B100R0	0.062 ± 0.000 a	0.095 ± 0.003 b	0.061 ± 0.002 a	0.0089 ± 0.0001 a	0.026 ± 0.001 a

B0R100, B10R90, B25R75 B50R50, B75R25, and B100R0: indicate the percentage of blue (B) and red (R) light; DW: dry weight. All values in the table are expressed as mean ± standard error (*n* = 3). Means with different letters are significantly different at the *p* < 0.05 level according to Tukey’s honestly significant difference test.

**Table 6:** Effect of different ratios of blue–red light in LED lighting on the growth parameters of kale microgreens.

Treatment	Hypocotyl Length, cm	Root Length, cm	Leaf Area, cm^2^	Shoot Fresh Weight, mg	Shoot Dry Weight, mg	Root Fresh Weight, mg	Root Dry Weight, mg	Shoot-to-Root Ratio
B0R100	4.92 ± 0.20 a	10.00 ± 0.36 a	3.44 ± 0.20 a	109.65 ± 6.58 ab	6.24 ± 0.62 ab	20.80 ± 1.96 b	1.35 ± 0.14 c	4.63 ± 0.07 b
B10R90	3.64 ± 0.24 b	8.45 ± 1.26 a	3.1 ± 0.25 a	112.38 ± 5.92 ab	7.23 ± 0.15 a	28.65 ± 0.89 a	1.72 ± 0.04 a	4.20 ± 0.13 c
B25R75	3.67 ± 0.12 b	9.04 ± 1.85 a	3.0 ± 0.30 a	116.19 ± 6.51 ab	6.69 ± 0.12 ab	28.02 ± 2.94 a	1.61 ± 0.02 ab	4.15 ± 0.14 c
B50R50	3.37 ± 0.13 b	9.29 ± 0.83 a	3.2 ± 0.29 a	119.42 ± 6.65 a	6.93 ± 0.26 a	28.91 ± 2.06 a	1.67 ± 0.14 ab	4.15 ± 0.26 c
B75R25	3.38 ± 0.16 b	8.2 ± 1.00 a	2.9 ± 0.25 a	100.14 ± 1.80 b	5.82 ± 0.14 b	23.01 ± 0.88 b	1.44 ± 0.03 bc	4.04 ± 0.13 c
B100R0	5.18 ± 0.14 a	9.9 ± 0.85 a	3.1 ± 0.17 a	125.16 ± 8.64 a	6.71 ± 0.61 ab	22.60 ± 1.01 b	1.29 ± 0.09 c	5.18 ± 0.16 a

B0R100, B10R90, B25R75 B50R50, B75R25, and B100R0: indicate the percentage of blue (B) and red (R) light. All values in the table are expressed as mean ± standard error (*n* = 3). Means with different letters are significantly different at the *p* < 0.05 level according to Tukey’s honestly significant difference test.

**Table 7:** Blue (B) and red (R) LED light combinations and total photon flux densities (TPFDs).

Treatments	Treatment Code	660 nm LED	447 nm LED
		μmol m^−^^2^ s^−^^1^	
0%B:100%R	B0R100	250	0
10%B:90%R	B10R90	225	25
25%B:75%R	B25R75	187	63
50%B:50%R	B50R50	125	125
75%B:25%R	B75R25	63	187
100%B:0%R	B100R0	0	250

## Data Availability

Not applicable.

## Supplementary Materials

The following are available online at https://www.mdpi.com/article/10.3390/plants10040801/s1. Table S1: Effect of different ratios of blue and red LED lighting on the bioconcentration factor of Brassicaceae microgreens. Table S2. Effect of different ratios of blue and red LED lighting on the bioconcentration factor of Brassicaceae microgreens. Table S3. Eigenvalue, factor scores, and contribution of the first three principal component axes to variation in Brassicaceae microgreens under different ratios of blue and red LED lighting. Table S4. Correlation matrix (Pearson (n)): mustard. Table S5. Correlation matrix (Pearson (n)): kale.
