# Supplementary material for: Effect of Different Ratios of Blue and Red LED Light on Brassicaceae Microgreens under a Controlled Environment
Source: Plants (Basel). 2021 Apr 19;10(4):801. doi: 10.3390/plants10040801 (PMC8073284; doi:10.3390/plants10040801)
Supplement: Supplementary file 1 [file plants-10-00801-s001.zip › plants-1158995-supplementary.pdf]

Table S1. Effect of different blue - red light ratio in LED lighting on the bioconcentration factor of mustard microgreens.

Treatment	BCF_P	BCF_K	BCF_{Ca}	BCF_{Mg}	BCF_S	BCF_{Mn}	BCF_{Fe}	BCF_{Zn}	BCF_{Cu}
B0R100	11.66 ± 0.07 d	5.59 ± 0.04 d	2.94 ± 0.04 b	1.19 ± 0.03 d	4.55 ± 0.13 d	11.67 ± 0.12 c	3.27 ± 0.01 c	4.93 ± 0.03 c	11.47 ± 0.12 c
B10R90	14.76 ± 0.23 c	7.65 ± 0.09 c	2.55 ± 0.06 c	1.58 ± 0.06 cd	5.54 ± 0.03 b	11.80 ± 0.22 c	3.04 ± 0.03 cd	5.66 ± 0.04 b	11.70 ± 0.08 c
B25R75	20.08 ± 0.19 a	9.78 ± 0.03 a	3.98 ± 0.02 a	2.39 ± 0.01 a	7.13 ± 0.06 a	12.97 ± 0.12 b	3.85 ± 0.03 b	6.60 ± 0.05 a	17.41 ± 0.12 b
B50R50	18.68 ± 0.12 b	8.80 ± 0.14 b	3.74 ± 0.03 a	2.04 ± 0.03 b	7.07 ±0.04 a	15.17 ± 0.13 a	4.39 ± 0.03 a	5.58 ± 0.01 b	23.51 ± 0.13 a
B75R25	13.60 ± 0.16 c	6.48 ± 0.03 c	2.59 ± 0.02 c	1.67 ± 0.02 c	4.93 ± 0.04 c	10.23 ± 0.15 d	3.05 ± 0.03 cd	5.16 ± 0.05 c	11.35 ± 0.08 c
B100R0	11.44 ± 0.15d	5.86 ± 0.05 d	2.35 ± 0.02 cd	1.45 ± 0.03 cd	4.23 ± 0.03 d	8.86 ± 0.08 e	2.42 ± 0.02 d	4.04 ± 0.03 d	9.66 ± 0.14 d

B0R100, B10R90, B25R75, B50R50, B75R25, B100R0 – percentage of blue (B) and red (R) light. BCF - bioconcentration factor; P – phosphorus, K - potassium, Ca – calcium, Mg – magnesium, S – sulfur, Fe – iron, Zn – zinc, Mn – manganese, Cu – copper, B – boron. All values in the Table are expressed as mean ± standard error (n = 3). Means with different letters are significantly different at the $P < 0.05$ level by Tukey's honestly significant difference test.

Table S2. Effect of different blue - red light ratio in LED lighting LED lighting on the translocation factor of mustard microgreens.

Treatment	TF_P	TF_K	TF_{Ca}	TF_{Mg}	TF_S	TF_{Mn}	TF_{Fe}	TF_{Zn}	TF_{Cu}
B0R100	0.96 ± 0.01 a	0.82 ± 0.01 a	2.08 ± 0.01 c	2.85 ± 0.05 a	1.49 ± 0.06 a	1.73 ± 0.01 c	0.19 ± 0.003 f	0.51 ± 0.009 cd	0.28 ± 0.013 c
B10R90	0.76 ± 0.02 b	0.64 ± 0.01 c	2.49 ± 0.06 a	2.30 ± 0.09 c	1.29 ± 0.01 d	1.76 ± 0.04 c	0.26 ± 0.006 c	0.49 ± 0.001 d	0.30 ± 0.005 b
B25R75	0.78 ± 0.02 b	0.67 ± 0.01 c	2.18 ± 0.04 c	2.08 ± 0.05 d	1.33 ± 0.02 cd	2.33 ± 0.07 a	0.24 ± 0.003 d	0.53 ± 0.004 c	0.27 ± 0.003 c
B50R50	0.94 ± 0.02 a	0.77 ± 0.01 b	2.51 ± 0.03 a	2.57 ± 0.07 b	1.39 ± 0.01 bc	2.01 ± 0.04 b	0.21 ± 0.002 e	0.70 ± 0.004 a	0.21 ± 0.002 d
B75R25	0.96 ± 0.03 a	0.79 ± 0.01 ab	2.61 ± 0.04 a	2.32 ± 0.07 c	1.50 ± 0.03 a	2.26 ± 0.05 a	0.28 ± 0.006 b	0.62 ± 0.008 b	0.36 ± 0.006 a
B100R0	0.92 ± 0.03 a	0.79 ± 0.02 ab	2.35 ± 0.07 b	2.26 ± 0.08 cd	1.44 ± 0.04 ab	2.22 ± 0.07 a	0.29 ± 0.004 a	0.64 ± 0.015 b	0.35 ± 0.008 a

B0R100, B10R90, B25R75, B50R50, B75R25, B100R0 – percentage of blue (B) and red (R) light. TF - translocation factor; N – nitrogen, P – phosphorus, K - potassium, Ca – calcium, Mg – magnesium, S – sulfur, Fe – iron, Zn – zinc, Mn – manganese, Cu – copper, B – boron. All values in the Table are expressed as mean ± standard error (n = 3). Means with different letters are significantly different at the $P < 0.05$ level by Tukey's honestly significant difference test.

Table S3. Eigenvalue, factor scores, and contribution of the first five principal component axes to variation in mustard microgreens under different blue - red light ratio in LED lighting.

Factors	F1	F2	F3	F4	F5
Eigenvalue	13.632	4.728	2.889	1.907	1.515
Variability (%)	48.686	16.885	10.317	6.812	5.409
Cumulative variability (%)			88.109		
Nitrogen	-0.222	-0.162	0.080	-0.281	-0.007
Phosphorus	0.237	-0.132	-0.110	0.077	-0.226
Potassium	0.261	0.079	0.045	-0.015	0.009
Calcium	0.213	-0.132	-0.165	0.185	-0.300
Magnesium	0.253	-0.091	-0.027	0.099	-0.164
Sulfur	0.224	-0.104	-0.152	0.005	-0.311
Manganese	0.251	-0.034	0.037	0.008	-0.169
Iron	0.186	-0.084	0.185	-0.323	0.083
Copper	0.252	-0.069	0.060	-0.143	-0.055
Zinc	0.243	-0.099	-0.051	-0.184	-0.115
Boron	0.123	0.141	-0.235	0.323	0.168
Nitrates	-0.201	-0.101	0.093	-0.420	-0.066
Nitrites	-0.123	-0.252	-0.028	0.422	0.205
Hypocotyl length	-0.052	0.428	-0.033	0.108	0.088
Root length	0.167	0.169	-0.098	-0.270	0.002
Leaf area	0.096	0.297	0.253	0.074	0.199
Shoot fresh weight	0.048	0.326	0.296	0.042	-0.073
Shoot dry weight	-0.015	0.167	0.384	0.042	-0.237
Root fresh weight	-0.030	-0.066	0.412	0.298	-0.243
Root dry weight	-0.123	-0.180	0.344	0.204	-0.271
Chlorophyll index	0.048	-0.418	0.065	0.087	0.177
Flavonols index	-0.055	-0.367	0.094	-0.010	0.315
Anthocyanin reflectance index (ARI1)	-0.261	-0.027	0.035	0.037	-0.125
Carotenoid reflectance index (CRI2)	-0.258	0.022	-0.008	0.026	-0.158

Normalized difference vegetation index (NDVI)	-0.233	-0.024	0.034	-0.082	-0.194
Photochemical reflectance index (PRI)	0.207	-0.092	0.265	0.014	0.289
Plant senescence reflectance index (PSRI)	-0.210	-0.009	-0.227	-0.050	-0.273
Water band index (WBI)	-0.192	0.080	-0.294	0.104	-0.009

Table S4. Correlation matrix (Pearson (n)) of mustard microgreens under different blue - red light ratio in LED lighting.

Variables	Hypocotyl length	Root length	Leaf area	Shoot fresh weight	Shoot dry weight	Root fresh weight	Root dry weight	Magnesium	Potassium	Calcium	Manganese	Iron	Copper	Zinc	Boron	Phosphorus	Sulfur	Nitrogen	Nitrates	Nitrites	Chlorophyll index	Flavonols index	Anthocyanin reflectance index (ARI1)	Carotenoid reflectance index (CRI2)	Normalized difference vegetation index (NDVI)	Photochemical reflectance index (PRI)	Plant senescence reflectance index (PSRI)	Water band index (WBI)
Hypocotyl length	**1**	0.200	**0.487**	**0.572**	0.328	-0.089	-0.321	-0.367	-0.007	-0.416	-0.312	-0.316	-0.345	-0.384	0.352	-0.465	-0.391	-0.258	-0.191	-0.292	**-0.834**	**-0.645**	0.128	0.225	0.085	-0.326	0.147	0.308
Root length	0.200	**1**	0.293	0.302	0.022	-0.345	**-0.640**	0.461	**0.649**	0.363	**0.542**	0.405	**0.506**	**0.574**	0.387	0.418	0.447	**-0.540**	-0.377	**-0.695**	-0.219	-0.277	**-0.617**	**-0.551**	-0.398	0.297	-0.374	-0.441
Leaf area	**0.487**	0.293	**1**	**0.689**	0.284	0.123	-0.210	0.167	0.442	-0.068	0.335	0.122	0.216	0.021	0.156	0.052	-0.051	**-0.496**	-0.390	**-0.488**	-0.409	-0.463	-0.386	-0.341	-0.349	0.410	**-0.656**	-0.301
Shoot fresh weight	**0.572**	0.302	**0.689**	**1**	**0.601**	0.230	-0.004	0.043	0.315	-0.148	0.173	0.132	0.113	-0.029	0.155	-0.098	-0.082	-0.309	-0.202	-0.447	**-0.513**	**-0.512**	-0.144	-0.098	-0.147	0.179	-0.337	-0.149
Shoot dry weight	0.328	0.022	0.284	**0.601**	**1**	0.362	0.449	-0.107	0.086	-0.245	-0.047	0.118	-0.044	-0.138	-0.054	-0.235	-0.128	0.000	0.047	-0.163	-0.302	-0.210	0.088	0.051	0.034	0.114	0.067	-0.230
Root fresh weight	-0.089	-0.345	0.123	0.230	0.362	**1**	**0.634**	0.001	-0.078	-0.018	-0.018	0.003	-0.036	-0.139	-0.283	-0.076	-0.155	0.060	-0.013	0.269	0.131	0.092	0.268	0.210	0.216	0.152	-0.162	-0.257
Root dry weight	-0.321	**-0.640**	-0.210	-0.004	0.449	**0.634**	**1**	-0.286	-0.467	-0.231	-0.288	-0.212	-0.346	-0.406	-0.433	-0.274	-0.280	**0.488**	0.393	0.447	0.317	0.397	**0.513**	0.450	0.465	-0.151	0.246	0.023
Magnesium	-0.367	0.461	0.167	0.043	-0.107	0.001	-0.286	**1**	**0.866**	**0.915**	**0.948**	**0.562**	**0.859**	**0.860**	0.367	**0.963**	**0.898**	**-0.745**	**-0.715**	-0.278	0.325	-0.123	**-0.845**	**-0.848**	**-0.732**	**0.671**	**-0.659**	**-0.647**
Potassium	-0.007	**0.649**	0.442	0.315	0.086	-0.078	-0.467	**0.866**	**1**	**0.655**	**0.858**	**0.718**	**0.883**	**0.843**	**0.473**	**0.753**	**0.734**	**-0.828**	**-0.736**	**-0.513**	0.021	-0.325	**-0.944**	**-0.924**	**-0.828**	**0.737**	**-0.755**	**-0.690**
Calcium	-0.416	0.363	-0.068	-0.148	-0.245	-0.018	-0.231	**0.915**	**0.655**	**1**	**0.837**	0.303	**0.705**	**0.758**	0.396	**0.967**	**0.915**	**-0.680**	**-0.682**	-0.153	0.321	-0.103	**-0.673**	**-0.664**	**-0.589**	0.410	-0.414	-0.449
Manganese	-0.312	**0.542**	0.335	0.173	-0.047	-0.018	-0.288	**0.948**	**0.858**	**0.837**	**1**	**0.538**	**0.849**	**0.807**	0.269	**0.917**	**0.814**	**-0.731**	**-0.642**	**-0.479**	0.213	-0.195	**-0.851**	**-0.831**	**-0.689**	**0.682**	**-0.731**	**-0.731**
Iron	-0.316	0.405	0.122	0.132	0.118	0.003	-0.212	**0.562**	**0.718**	0.303	**0.538**	**1**	**0.854**	**0.821**	0.064	**0.469**	**0.531**	-0.254	-0.164	-0.374	0.265	0.064	**-0.667**	**-0.710**	**-0.605**	**0.731**	**-0.592**	**-0.669**
Copper	-0.345	**0.506**	0.216	0.113	-0.044	-0.036	-0.346	**0.859**	**0.883**	**0.705**	**0.849**	**0.854**	**1**	**0.963**	0.251	**0.826**	**0.820**	**-0.605**	**-0.515**	-0.454	0.253	-0.120	**-0.872**	**-0.884**	**-0.796**	**0.762**	**-0.726**	**-0.714**
Zinc	-0.384	**0.574**	0.021	-0.029	-0.138	-0.139	-0.406	**0.860**	**0.843**	**0.758**	**0.807**	**0.821**	**0.963**	**1**	0.285	**0.851**	**0.886**	**-0.561**	**-0.476**	-0.426	0.279	-0.085	**-0.835**	**-0.837**	**-0.726**	**0.628**	**-0.572**	**-0.628**
Boron	0.352	0.387	0.156	0.155	-0.054	-0.283	-0.433	0.367	**0.473**	0.396	0.269	0.064	0.251	0.285	**1**	0.336	0.390	**-0.665**	**-0.706**	-0.010	-0.140	-0.217	**-0.473**	-0.415	**-0.504**	0.184	-0.230	-0.042
Phosphorus	-0.465	0.418	0.052	-0.098	-0.235	-0.076	-0.274	**0.963**	**0.753**	**0.967**	**0.917**	**0.469**	**0.826**	**0.851**	0.336	**1**	**0.945**	**-0.672**	**-0.642**	-0.264	0.356	-0.090	**-0.782**	**-0.777**	**-0.673**	**0.547**	**-0.560**	**-0.546**
Sulfur	-0.391	0.447	-0.051	-0.082	-0.128	-0.155	-0.280	**0.898**	**0.734**	**0.915**	**0.814**	**0.531**	**0.820**	**0.886**	0.390	**0.945**	**1**	**-0.602**	**-0.554**	-0.308	0.244	-0.163	**-0.734**	**-0.725**	**-0.659**	0.425	-0.388	-0.435
Nitrogen	-0.258	**-0.540**	**-0.496**	-0.309	0.000	0.060	**0.488**	**-0.745**	**-0.828**	**-0.680**	**-0.731**	-0.254	**-0.605**	**-0.561**	**-0.665**	**-0.672**	**-0.602**	**1**	**0.964**	0.359	0.132	0.455	**0.807**	**0.749**	**0.754**	**-0.487**	**0.618**	0.447
Nitrates	-0.191	-0.377	-0.390	-0.202	0.047	-0.013	0.393	**-0.715**	**-0.736**	**-0.682**	**-0.642**	-0.164	**-0.515**	**-0.476**	**-0.706**	**-0.642**	**-0.554**	**0.964**	**1**	0.097	-0.012	0.319	**0.727**	**0.696**	**0.735**	**-0.482**	**0.570**	0.364
Nitrites	-0.292	**-0.695**	**-0.488**	-0.447	-0.163	0.269	0.447	-0.278	**-0.513**	-0.153	**-0.479**	-0.374	-0.454	-0.426	-0.010	-0.264	-0.308	0.359	0.097	**1**	**0.533**	**0.583**	0.468	0.362	0.241	-0.130	0.310	0.397
Chlorophyll index	**-0.834**	-0.219	-0.409	**-0.513**	-0.302	0.131	0.317	0.325	0.021	0.321	0.213	0.265	0.253	0.279	-0.140	0.356	0.244	0.132	-0.012	**0.533**	**1**	**0.839**	-0.136	-0.237	-0.124	0.422	-0.249	-0.317
Flavonols index	**-0.645**	-0.277	-0.463	**-0.512**	-0.210	0.092	0.397	-0.123	-0.325	-0.103	-0.195	0.064	-0.120	-0.085	-0.217	-0.090	-0.163	0.455	0.319	**0.583**	**0.839**	**1**	0.205	0.098	0.193	0.192	0.020	-0.161
Anthocyanin reflectance index (ARI1)	0.128	**-0.617**	-0.386	-0.144	0.088	0.268	**0.513**	**-0.845**	**-0.944**	**-0.673**	**-0.851**	**-0.667**	**-0.872**	**-0.835**	**-0.473**	**-0.782**	**-0.734**	**0.807**	**0.727**	0.468	-0.136	0.205	**1**	**0.981**	**0.892**	**-0.737**	**0.761**	**0.646**
Carotenoid reflectance index (CRI2)	0.225	**-0.551**	-0.341	-0.098	0.051	0.210	0.450	**-0.848**	**-0.924**	**-0.664**	**-0.831**	**-0.710**	**-0.884**	**-0.837**	-0.415	**-0.777**	**-0.725**	**0.749**	**0.696**	0.362	-0.237	0.098	**0.981**	**1**	**0.914**	**-0.820**	**0.768**	**0.680**
Normalized difference vegetation index (NDVI)	0.085	-0.398	-0.349	-0.147	0.034	0.216	0.465	**-0.732**	**-0.828**	**-0.589**	**-0.689**	**-0.605**	**-0.796**	**-0.726**	**-0.504**	**-0.673**	**-0.659**	**0.754**	**0.735**	0.241	-0.124	0.193	**0.892**	**0.914**	**1**	**-0.728**	**0.660**	**0.472**
Photochemical reflectance index (PRI)	-0.326	0.297	0.410	0.179	0.114	0.152	-0.151	**0.671**	**0.737**	0.410	**0.682**	**0.731**	**0.762**	**0.628**	0.184	**0.547**	0.425	**-0.487**	**-0.482**	-0.130	0.422	0.192	**-0.737**	**-0.820**	**-0.728**	**1**	**-0.866**	**-0.804**
Plant senescence reflectance index (PSRI)	0.147	-0.374	**-0.656**	-0.337	0.067	-0.162	0.246	**-0.659**	**-0.755**	-0.414	**-0.731**	**-0.592**	**-0.726**	**-0.572**	-0.230	**-0.560**	-0.388	**0.618**	**0.570**	0.310	-0.249	0.020	**0.761**	**0.768**	**0.660**	**-0.866**	**1**	**0.705**
Water band index (WBI)	0.308	-0.441	-0.301	-0.149	-0.230	-0.257	0.023	**-0.647**	**-0.690**	-0.449	**-0.731**	**-0.669**	**-0.714**	**-0.628**	-0.042	**-0.546**	-0.435	0.447	0.364	0.397	-0.317	-0.161	**0.646**	**0.680**	**0.472**	**-0.804**	**0.705**	**1**

Values in bold are different from 0 with a significance level alpha=0.05.

Table S5. Effect of different blue - red light ratio in LED lighting on the bioconcentration factor of kale microgreens.

Treatment	BCF_P	BCF_K	BCF_{Ca}	BCF_{Mg}	BCF_S	BCF_{Mn}	BCF_{Fe}	BCF_{Zn}	BCF_{Cu}
B0R100	19.53 ± 1.17 ab	10.04 ± 0.59 ab	2.86 ± 0.25 ab	5.80 ± 0.28 a	14.23 ± 1.14 b	8.71 ± 0.25 bc	2.86 ± 0.08 bc	5.32 ± 0.26 ab	13.01 ± 0.46 b
B10R90	18.26 ± 1.81 ab	8.66 ± 0.48 c	2.53 ± 0.13 b	5.44 ± 0.64 ab	13.13 ± 0.88 b	8.07 ± 0.41 cd	2.43 ± 0.07 d	5.21 ± 0.63 ab	12.94 ± 1.01 b
B25R75	19.09 ± 0.20 ab	9.88 ± 0.14 b	2.93 ± 0.07 ab	5.74 ± 0.21 ab	14.04 ± 0.12 b	8.22 ± 0.07 c	2.54 ± 0.02 d	5.10 ± 0.02 b	12.29 ± 0.07 b
B50R50	18.14 ± 0.18 ab	9.07 ± 0.12 bc	2.74 ± 0.04 ab	5.29 ± 0.11 bc	13.71 ± 0.06 b	8.51 ± 0.12 bc	2.66 ± 0.03 c	5.04 ± 0.03 b	13.31 ± 0.16 ab
B75R25	21.74 ± 0.43 a	12.13 ± 0.17 a	3.54 ± 0.07 a	5.33 ± 0.21 bc	16.30 ± 0.02 a	10.66 ± 0.18 a	3.29 ± 0.04 a	6.51 ± 0.07 a	15.17 ± 0.18 a
B100R0	17.81 ± 0.30 ab	8.78 ± 0.15 c	3.03 ± 0.08 a	4.39 ± 0.19 c	14.15 ± 0.10 b	8.91 ± 0.15 ab	2.98 ± 0.04 b	5.45 ± 0.03 ab	13.29 ± 0.07 ab

B0R100, B10R90, B25R75, B50R50, B75R25, B100R0 – percentage of blue (B) and red (R) light. BCF - bioconcentration factor; P – phosphorus, K - potassium, Ca – calcium, Mg – magnesium, S – sulfur, Fe – iron, Zn – zinc, Mn – manganese, Cu – copper, B – boron. All values in the Table are expressed as mean ± standard error (n = 3). Means with different letters are significantly different at the $P < 0.05$ level by Tukey's honestly significant difference test.

Table S6. Effect of different blue - red light ratio in LED lighting on the translocation factor of kale microgreens.

Treatment	TF_P	TF_K	TF_{Ca}	TF_{Mg}	TF_S	TF_{Mn}	TF_{Fe}	TF_{Zn}	TF_{Cu}
B0R100	0.88 ± 0.07 a	0.80 ± 0.06 ab	3.14 ± 0.29 bc	1.07 ± 0.06 b	0.58 ± 0.07 c	3.51 ± 0.12 ab	0.31 ± 0.01 c	0.53 ± 0.03 a	0.22 ± 0.01 c
B10R90	0.91 ± 0.12 a	0.87 ± 0.07 a	3.66 ± 0.23 a	1.11 ± 0.17 b	0.67 ± 0.04 bc	3.56 ± 0.25 ab	0.40 ± 0.02 a	0.54 ± 0.09 a	0.23 ± 0.03 c
B25R75	1.01 ± 0.02 a	0.84 ± 0.02 ab	3.50 ± 0.11 ab	1.12 ± 0.05 b	0.75 ± 0.02 ab	3.82 ± 0.05 a	0.37 ± 0.01 ab	0.59 ± 0.01 a	0.27 ± 0.01 bc
B50R50	0.97 ± 0.02 a	0.84 ± 0.01 ab	3.48 ± 0.06 ab	1.12 ± 0.01 b	0.76 ± 0.02 ab	3.43 ± 0.01 bc	0.33 ± 0.02 bc	0.58 ± 0.05 a	0.30 ± 0.01 ab
B75R25	0.96 ± 0.04 a	0.73 ± 0.02 b	2.97 ± 0.13 c	1.31 ± 0.08 ab	0.79 ± 0.02 a	3.16 ± 0.10 c	0.38 ± 0.01 a	0.56 ± 0.01 a	0.32 ± 0.04 ab
B100R0	1.05 ± 0.02 a	0.91 ± 0.03 a	3.11 ± 0.11 bc	1.40 ± 0.07 a	0.83 ± 0.01 a	3.65 ± 0.04 ab	0.34 ± 0.02 bc	0.59 ± 0.02 a	0.35 ± 0.01 a

B0R100, B10R90, B25R75, B50R50, B75R25, B100R0 – percentage of blue (B) and red (R) light. TF - translocation factor; N – nitrogen, P – phosphorus, K - potassium, Ca – calcium, Mg – magnesium, S – sulfur, Fe – iron, Zn – zinc, Mn – manganese, Cu – copper, B – boron. All values in the Table are expressed as mean ± standard error (n = 3). Means with different letters are significantly different at the $P < 0.05$ level by Tukey's honestly significant difference test.

Table S7. Eigenvalue, factor scores, and contribution of the first six principal component axes to variation in kale microgreens under different blue - red light ratio LED lighting.

Factors	F1	F2	F3	F4	F5	F6
Eigenvalue	8.946	5.513	3.766	2.330	1.910	1.546
Variability (%)	31.948	19.690	13.452	8.321	6.820	5.523
Cumulative variability (%)			85.754			
Nitrogen	-0.094	0.272	0.196	0.256	-0.194	0.193
Phosphorus	0.287	0.069	0.077	0.015	0.246	-0.133
Potassium	0.251	-0.090	0.242	0.107	0.166	-0.059
Calcium	0.144	0.312	0.120	0.002	0.286	-0.099
Magnesium	0.238	-0.034	0.168	0.235	0.194	-0.059
Sulfur	0.300	0.127	-0.050	-0.133	0.023	-0.051
Manganese	0.256	-0.061	0.279	0.036	-0.059	0.092
Iron	0.193	-0.019	-0.326	0.045	-0.242	0.139
Copper	0.295	0.067	-0.052	-0.201	0.015	0.162
Zinc	0.283	0.037	-0.127	-0.046	-0.076	0.093
Boron	0.200	0.103	0.068	0.001	-0.213	0.347
Nitrates	-0.100	0.266	0.197	0.262	-0.196	0.192
Nitrites	0.230	0.053	-0.146	-0.392	0.083	0.017
Hypocotyl length	0.102	-0.237	0.326	0.106	-0.222	0.089
Root length	0.034	-0.135	0.326	-0.116	0.021	-0.172
Leaf area	-0.092	-0.177	0.298	-0.304	0.002	0.011
Shoot fresh weight	0.063	0.174	0.367	-0.180	-0.154	0.023
Shoot dry weight	-0.077	0.283	0.265	-0.087	-0.134	0.128
Root fresh weight	-0.119	0.354	0.001	-0.042	0.142	0.007
Root dry weight	-0.169	0.320	-0.054	-0.075	0.060	-0.056
Chlorophyll index	0.028	0.323	-0.042	-0.305	-0.221	-0.179
Flavonols index	0.261	0.124	-0.150	-0.061	-0.287	0.026
Anthocyanin reflectance index (ARI1)	-0.150	0.030	-0.087	-0.126	0.239	0.583
Carotenoid reflectance index (CRI2)	-0.229	-0.123	-0.013	-0.160	-0.049	0.231
Normalized difference vegetation index (NDVI)	-0.236	-0.127	0.032	-0.116	-0.104	-0.105
Photochemical reflectance index (PRI)	-0.143	0.211	0.013	-0.053	-0.217	-0.438
Plant senescence reflectance index (PSRI)	-0.058	0.247	0.006	0.188	0.443	0.073
Water band index (WBI)	0.094	0.048	-0.204	0.476	-0.189	-0.119

Table S8. Correlation matrix (Pearson (n)) of kale microgreens under different blue - red light ratio LED lighting.

Variables	Hypocotyl length	Root length	Leaf area	Shoot fresh weight	Shoot dry weight	Root fresh weight	Root dry weight	Magnesium	Potassium	Calcium	Manganese	Iron	Copper	Zinc	Boron	Phosphorus	Sulfur	Nitrogen	Nitrates	Nitrites	Chlorophyll index	Flavonols index	Anthocyanin reflectance index (ARI1)	Carotenoid reflectance index (CRI2)	Normalized difference vegetation index (NDVI)	Photochemical reflectance index (PRI)	Plant senescence reflectance index (PSRI)	Water band index (WBI)
Hypocotyl length	**1**	**0.494**	0.353	0.372	-0.050	**-0.652**	**-0.696**	0.422	**0.623**	-0.236	**0.749**	-0.074	0.070	0.132	0.161	0.132	0.003	-0.042	-0.038	-0.144	-0.464	0.029	-0.299	0.010	0.035	-0.346	**-0.480**	-0.036
Root length	**0.494**	**1**	**0.583**	0.301	0.029	-0.104	-0.293	0.126	0.327	-0.109	0.380	-0.339	0.052	-0.042	-0.020	0.092	-0.032	-0.067	-0.066	-0.029	-0.214	-0.240	-0.359	-0.180	-0.011	-0.207	-0.222	-0.336
Leaf area	0.353	**0.583**	**1**	0.221	0.127	-0.262	-0.187	-0.121	0.006	-0.320	0.059	**-0.511**	-0.183	-0.465	-0.040	-0.215	-0.324	-0.113	-0.105	-0.151	-0.153	**-0.488**	0.068	0.240	0.410	-0.069	-0.296	**-0.695**
Shoot fresh weight	0.372	0.301	0.221	**1**	**0.682**	0.214	0.168	0.118	0.334	0.458	**0.506**	-0.343	0.229	0.152	0.275	0.248	0.273	0.391	0.381	0.118	0.451	0.188	-0.142	-0.118	-0.047	0.191	0.073	-0.270
Shoot dry weight	-0.050	0.029	0.127	**0.682**	**1**	**0.644**	**0.671**	-0.043	-0.091	0.420	0.016	-0.402	-0.144	-0.227	0.214	-0.139	-0.115	**0.628**	**0.626**	-0.149	**0.520**	-0.010	0.109	0.126	-0.039	0.349	0.209	-0.214
Root fresh weight	**-0.652**	-0.104	-0.262	0.214	**0.644**	**1**	**0.885**	-0.297	-0.426	**0.500**	-0.402	-0.273	-0.164	-0.182	-0.091	-0.152	-0.086	**0.550**	**0.545**	-0.063	**0.527**	-0.106	0.262	-0.009	-0.056	0.419	**0.599**	-0.098
Root dry weight	**-0.696**	-0.293	-0.187	0.168	**0.671**	**0.885**	**1**	-0.389	**-0.534**	0.316	**-0.615**	-0.303	-0.322	-0.312	-0.158	-0.323	-0.256	0.414	0.415	-0.166	**0.565**	-0.152	0.269	0.148	0.130	**0.518**	**0.474**	-0.047
Magnesium	0.422	0.126	-0.121	0.118	-0.043	-0.297	-0.389	**1**	**0.895**	**0.480**	**0.714**	0.139	0.409	0.396	0.403	**0.762**	**0.475**	-0.132	-0.139	0.202	-0.257	0.324	-0.421	**-0.527**	**-0.540**	-0.383	-0.009	0.246
Potassium	**0.623**	0.327	0.006	0.334	-0.091	-0.426	**-0.534**	**0.895**	**1**	0.403	**0.846**	0.025	**0.491**	0.444	0.352	**0.782**	**0.516**	-0.237	-0.245	0.279	-0.248	0.306	-0.414	-0.435	**-0.488**	-0.436	-0.125	0.094
Calcium	-0.236	-0.109	-0.320	0.458	0.420	**0.500**	0.316	**0.480**	0.403	**1**	0.347	-0.094	0.429	0.285	0.254	**0.689**	**0.612**	0.304	0.286	0.388	**0.490**	0.344	-0.104	**-0.524**	**-0.517**	0.183	**0.560**	0.009
Manganese	**0.749**	0.380	0.059	**0.506**	0.016	-0.402	**-0.615**	**0.714**	**0.846**	0.347	**1**	0.176	**0.579**	**0.568**	0.462	**0.679**	**0.595**	-0.010	-0.022	0.342	-0.133	0.439	-0.361	-0.428	-0.396	-0.381	-0.230	-0.004
Iron	-0.074	-0.339	**-0.511**	-0.343	-0.402	-0.273	-0.303	0.139	0.025	-0.094	0.176	**1**	**0.584**	**0.749**	0.364	0.254	**0.556**	-0.225	-0.235	**0.494**	0.034	**0.721**	-0.167	-0.382	-0.362	-0.268	-0.292	0.435
Copper	0.070	0.052	-0.183	0.229	-0.144	-0.164	-0.322	0.409	**0.491**	0.429	**0.579**	**0.584**	**1**	**0.837**	**0.656**	**0.735**	**0.924**	-0.225	-0.247	**0.830**	0.287	**0.774**	-0.158	**-0.589**	**-0.647**	-0.378	-0.068	0.028
Zinc	0.132	-0.042	-0.465	0.152	-0.227	-0.182	-0.312	0.396	0.444	0.285	**0.568**	**0.749**	**0.837**	**1**	**0.482**	**0.634**	**0.822**	-0.250	-0.268	**0.685**	0.125	**0.784**	-0.271	**-0.551**	**-0.497**	-0.352	-0.099	0.286
Boron	0.161	-0.020	-0.040	0.275	0.214	-0.091	-0.158	0.403	0.352	0.254	0.462	0.364	**0.656**	**0.482**	**1**	0.435	**0.523**	0.188	0.176	0.313	0.266	**0.597**	-0.134	-0.368	**-0.606**	-0.285	-0.119	0.173
Phosphorus	0.132	0.092	-0.215	0.248	-0.139	-0.152	-0.323	**0.762**	**0.782**	**0.689**	**0.679**	0.254	**0.735**	**0.634**	0.435	**1**	**0.853**	-0.190	-0.208	**0.549**	0.126	**0.502**	-0.386	**-0.710**	**-0.638**	-0.244	0.134	0.120
Sulfur	0.003	-0.032	-0.324	0.273	-0.115	-0.086	-0.256	**0.475**	**0.516**	**0.612**	**0.595**	**0.556**	**0.924**	**0.822**	**0.523**	**0.853**	**1**	-0.150	-0.174	**0.798**	0.405	**0.801**	-0.337	**-0.708**	**-0.640**	-0.147	0.014	0.131
Nitrogen	-0.042	-0.067	-0.113	0.391	**0.628**	**0.550**	0.414	-0.132	-0.237	0.304	-0.010	-0.225	-0.225	-0.250	0.188	-0.190	-0.150	**1**	**1.000**	**-0.471**	0.259	-0.095	0.136	-0.095	0.005	0.413	0.428	0.093
Nitrates	-0.038	-0.066	-0.105	0.381	**0.626**	**0.545**	0.415	-0.139	-0.245	0.286	-0.022	-0.235	-0.247	-0.268	0.176	-0.208	-0.174	**1.000**	**1**	**-0.492**	0.245	-0.113	0.138	-0.080	0.019	0.414	0.423	0.095
Nitrites	-0.144	-0.029	-0.151	0.118	-0.149	-0.063	-0.166	0.202	0.279	0.388	0.342	**0.494**	**0.830**	**0.685**	0.313	**0.549**	**0.798**	**-0.471**	**-0.492**	**1**	0.405	**0.689**	-0.072	-0.352	-0.468	-0.249	-0.162	-0.136
Chlorophyll index	-0.464	-0.214	-0.153	0.451	**0.520**	**0.527**	**0.565**	-0.257	-0.248	**0.490**	-0.133	0.034	0.287	0.125	0.266	0.126	0.405	0.259	0.245	0.405	**1**	**0.476**	-0.123	-0.184	-0.181	**0.589**	0.060	-0.045
Flavonols index	0.029	-0.240	**-0.488**	0.188	-0.010	-0.106	-0.152	0.324	0.306	0.344	0.439	**0.721**	**0.774**	**0.784**	**0.597**	**0.502**	**0.801**	-0.095	-0.113	**0.689**	**0.476**	**1**	-0.363	**-0.515**	**-0.581**	-0.111	-0.247	0.430
Anthocyanin reflectance index (ARI1)	-0.299	-0.359	0.068	-0.142	0.109	0.262	0.269	-0.421	-0.414	-0.104	-0.361	-0.167	-0.158	-0.271	-0.134	-0.386	-0.337	0.136	0.138	-0.072	-0.123	-0.363	**1**	**0.504**	0.266	-0.210	0.308	-0.375
Carotenoid reflectance index (CRI2)	0.010	-0.180	0.240	-0.118	0.126	-0.009	0.148	**-0.527**	-0.435	**-0.524**	-0.428	-0.382	**-0.589**	**-0.551**	-0.368	**-0.710**	**-0.708**	-0.095	-0.080	-0.352	-0.184	**-0.515**	**0.504**	**1**	**0.506**	0.098	-0.187	-0.426
Normalized difference vegetation index (NDVI)	0.035	-0.011	0.410	-0.047	-0.039	-0.056	0.130	**-0.540**	**-0.488**	**-0.517**	-0.396	-0.362	**-0.647**	**-0.497**	**-0.606**	**-0.638**	**-0.640**	0.005	0.019	-0.468	-0.181	**-0.581**	0.266	**0.506**	**1**	0.354	-0.134	-0.375
Photochemical reflectance index (PRI)	-0.346	-0.207	-0.069	0.191	0.349	0.419	**0.518**	-0.383	-0.436	0.183	-0.381	-0.268	-0.378	-0.352	-0.285	-0.244	-0.147	0.413	0.414	-0.249	**0.589**	-0.111	-0.210	0.098	0.354	**1**	0.137	-0.080
Plant senescence reflectance index (PSRI)	**-0.480**	-0.222	-0.296	0.073	0.209	**0.599**	**0.474**	-0.009	-0.125	**0.560**	-0.230	-0.292	-0.068	-0.099	-0.119	0.134	0.014	0.428	0.423	-0.162	0.060	-0.247	0.308	-0.187	-0.134	0.137	**1**	0.030
Water band index (WBI)	-0.036	-0.336	**-0.695**	-0.270	-0.214	-0.098	-0.047	0.246	0.094	0.009	-0.004	0.435	0.028	0.286	0.173	0.120	0.131	0.093	0.095	-0.136	-0.045	0.430	-0.375	-0.426	-0.375	-0.080	0.030	**1**

Values in bold are different from 0 with a significance level alpha=0.05.
